# Supplementary material for: Overlapping Systemic Proteins in COVID-19 and Lung Fibrosis Associated with Tissue Remodeling and Inflammation
Source: Biomedicines. 2024 Dec 19;12(12):2893. doi: 10.3390/biomedicines12122893 (PMC11727205; doi:10.3390/biomedicines12122893)
Supplement: Supplementary file 1 [file biomedicines-12-02893-s001.zip › biomedicines-3343743-supplementary.pdf]

Supplementary material

Table S1. Patient characteristics including laboratory measurements

	**Moderate COVID-19 (n=8)**	**Severe COVID-19 (n=8)**	**Healthy subjects (n=7)**
Age (Mean ± SD)	57.1 ± 6.7	64.9 ± 13.8	55.1 ± 12.8
Male/Female (n, %)	5/3<br>(62.5% /37.5%)	7/1<br>(87.5%/ 12.5%)	5/2<br>(71% /29%)
Smoking history<br>- Never smokers (n, %)<br>- Ex-smokers (n, %)<br>- Current smokers (n, %)<br>- Unknown (n, %)	 6 (75%)<br>2 (25%)<br>-<br>-	 2 (25%)<br>-<br>1 (12.5%)<br>5 (62.5%)	 3 (42.9%)<br>2 (28.6%)<br>2 (28.6%)
Laboratory parameters (Mean ± SD)<br>-CRP (mg/L)<br>-Prokalcitonin (ug/L)<br>-Leukocytes (x10^9/L)<br>-Neutrofiles (x10^9/L)<br>-Monocytes (x10^9/L)<br>-Lymphocytes (x10^9/L)<br>-IL-6 (ng/L)	52.0 ± 52.2<br>0.37 ± 0.36<br>6.6 ± 2.9<br>6.6 ± 2.9<br>0.43 ± 0.37<br>0.91 ± 0.59<br>22.9 ± 38.5	36.6 ± 63.9<br>0.13 ± 0.06<br>6.4 ± 3.2<br>4.5 ± 2.4<br>0.58 ± 0.44<br>1.1 ± 0.39<br>25. 6 ± 40.4	-<br>-<br>-<br>-<br>-<br>-<br>-
Death during hospital visit (n, %)	0 (0%)	2 (25%)	NA

Table S2. Proteins with >1 NPX mean difference between COVID-19 groups and healthy group.

Protein	Healthy (mean NPX)	Moderate COVID-19 (mean NPX)	Severe COVID-19 (mean NPX)
ADA	7.736	6.587	6.699
ADGRG1	2.838	3.375	4.543
ANGPT1	10.147	8.511	7.426
CAIX	4.899	5.291	6.303
CASP-8	9.271	6.512	6.818
CCL17	11.825	7.984	8.429
CCL19	11.642	12.191	13.154
CCL23	10.949	11.671	9.860
CD244	10.570	7.467	12.410
CD4	5.957	4.880	7.401
CD40	13.064	11.182	5.342
CD40-L	10.081	4.733	4.459
CD5	10.072	7.131	7.260
CD70	4.326	4.867	5.372
CXCL1	11.329	9.070	9.166
CXCL10	10.782	12.847	13.831
CXCL13	9.401	9.815	11.019
CXCL5	13.921	9.777	9.094
CXCL9	8.009	8.388	9.913
DCN	4.849	5.013	5.913
EGF	9.822	6.211	5.830
FASLG	8.387	7.332	6.900
FGF2	4.177	1.640	1.978
GAL-9	8.422	8.882	9.520
GZMA	10.553	8.067	8.189
GZMB	9.288	5.362	6.342
GZMH	9.869	5.702	5.956
HGF	9.730	11.114	12.502
IL15	6.043	6.855	7.222
IL6	3.914	5.949	7.273

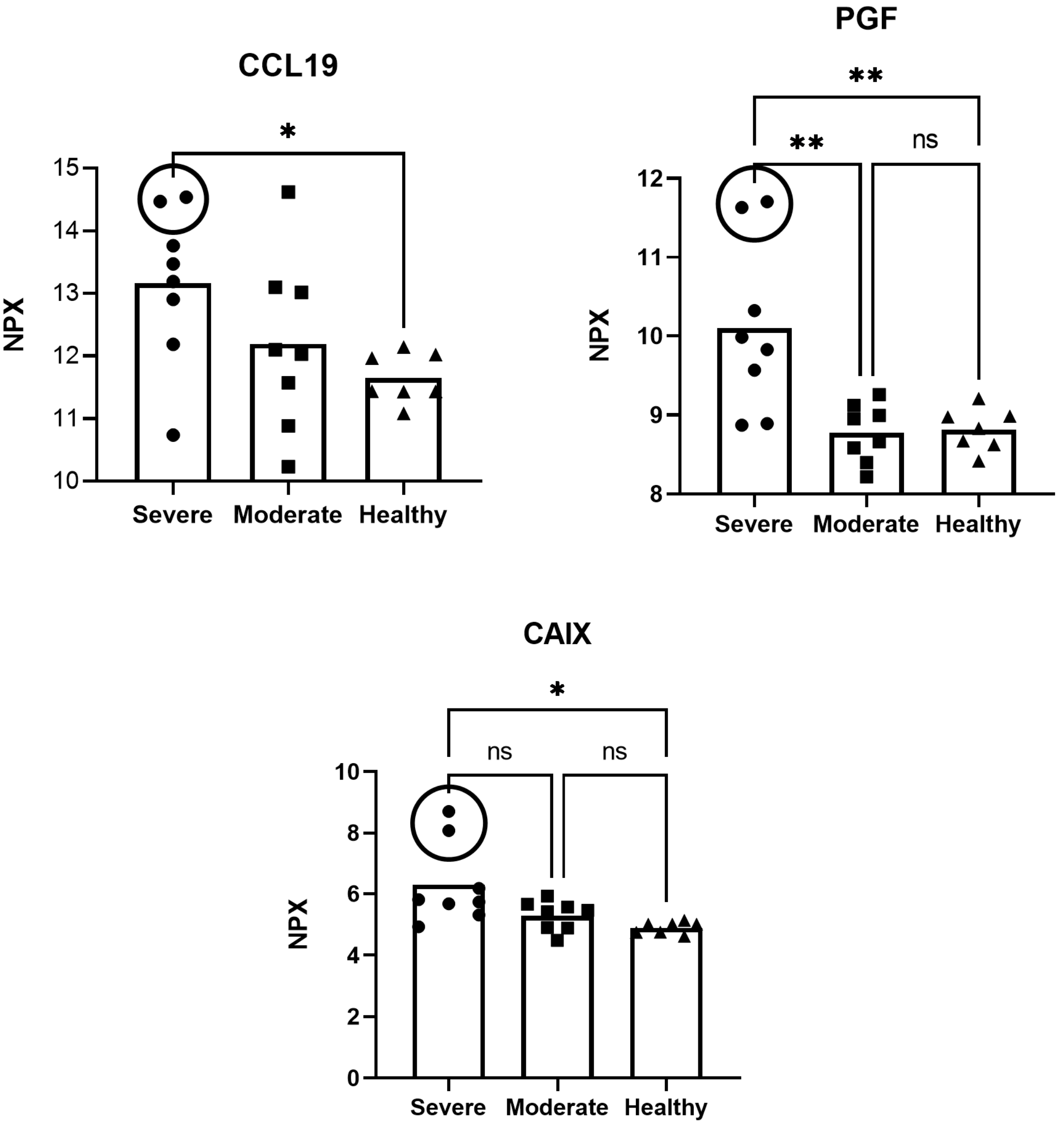

Figure S1. Elevated protein amount of CCL19, PGF, and CAIX in plasam from severe COVID-19 patients that died during hospital visit. Deceased patients during hospital visit (n=2, encircled). NPX=normalized protein expression. Moderate (n=8) and severe (n=8) COVID-19 patients, healthy individuals (n=7). One-way ANOVA with Tukey's multiple comparison test. *p<0.05, **p<0.01

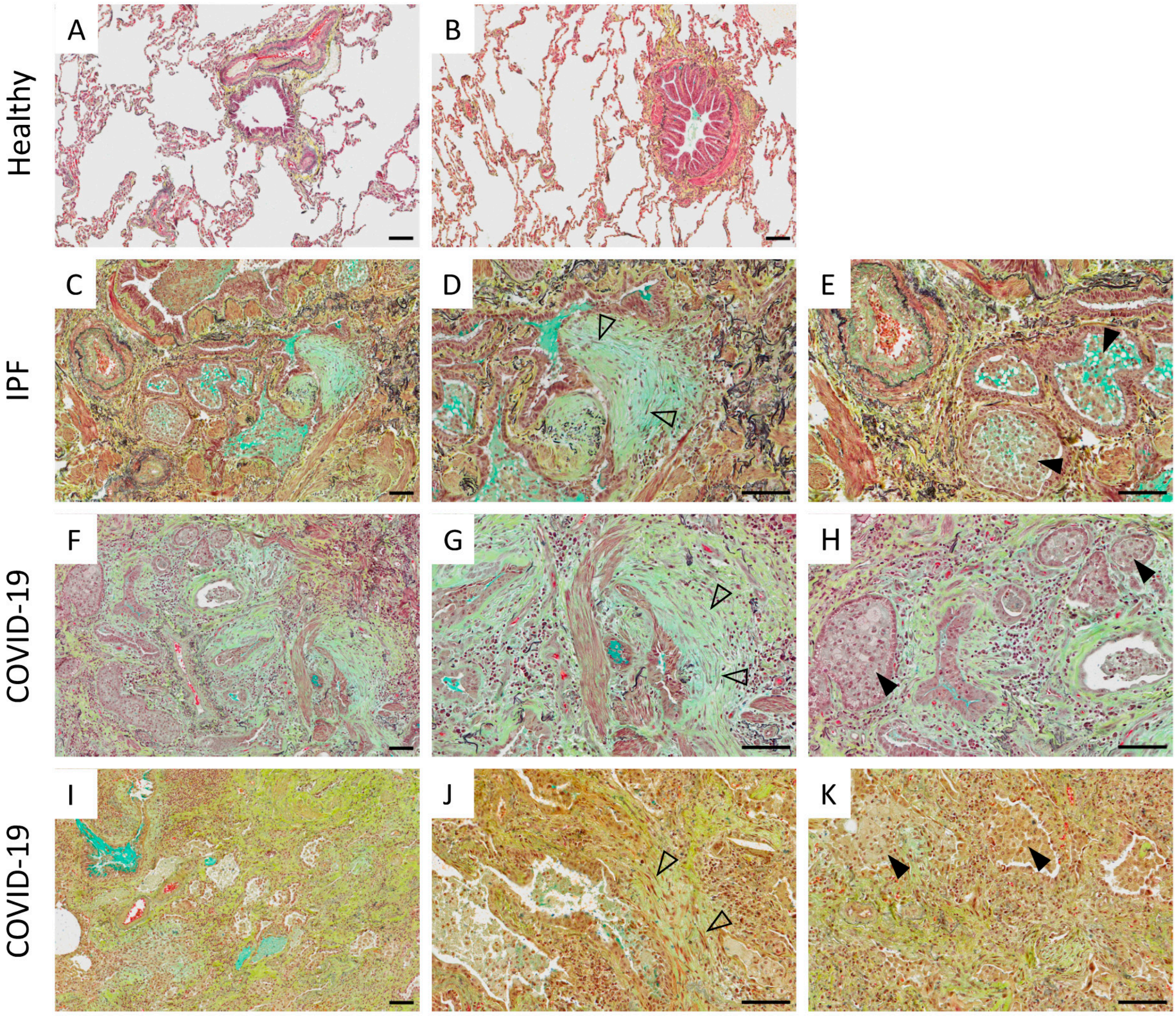

Figure S2. Lung tissue remodeling and shared histopathological features in COVID-19 and IPF. With modified Russel-Movat pentachrome stain visualizing different components of connective tissue, thin alveolar walls in healthy lung tissue were consisted mainly of collagens and elastic fibers (A,B). In COVID-19 lung tissue (F – patient 1, I ö patient 2), the parenchymal structure was disrupted and replaced by dense connective tissue rich in collagens and proteoglycans, mucus-filled epithelial structures, smooth muscle cell hyperplasia and accumulations of other cell types, similar to the histopathological features in IPF (C). Structures with spindle-shaped myofibroblasts similar to fibroblastic foci in IPF tissue (D, empty arrowheads) were found in COVID-19 lung tissue (G, J empty arrowheads). Alos, accumulations of macrophages were present in both COVID-19 (H, K black arrowheads) and IPF lung tissue No fibrin deposits were found with pentachrome staining in both COVID-19 and IPF lung tissue. Scale bars 100 µm (A-K). Legend for the pentachrome staining: yellow – collagens; green/blue – mucins/proteoglycans; black – elastic fibers; bright red – red blood cells; red – SMCs/fibrin; dark red – other cell types. Various shades of green can result also from colocalization of collagens (yellow) and proteoglycans (blue).

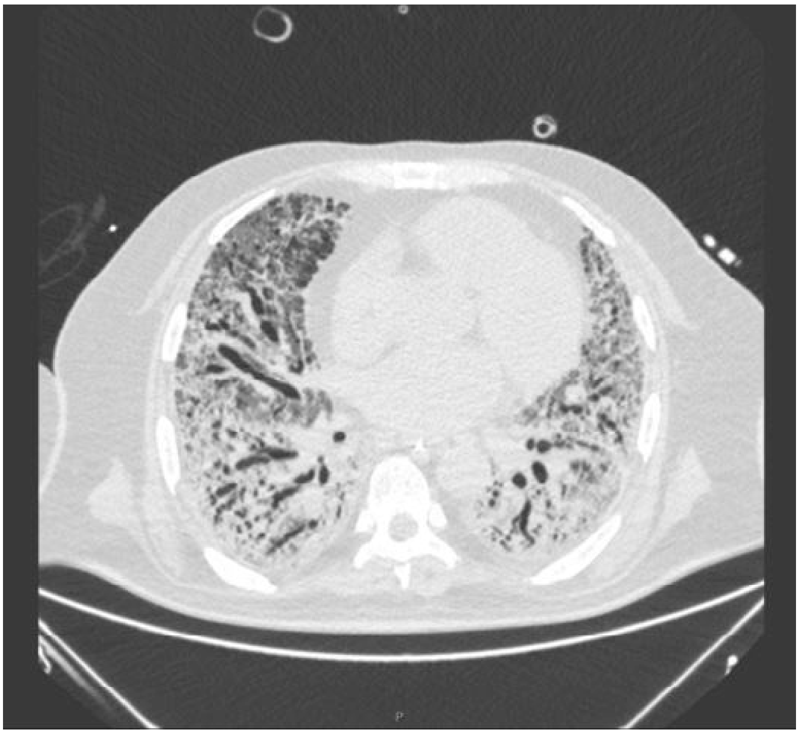

Figure S3. HRCT image of consolidated parenchymal changes. Bronchiectasis bilaterally, slightly more pronounced in the lower lobes. The picture is consistent with severe ARDS and fibrosis development. Lymphadenopathy in the upper mediastinum. In addition, several lymph nodes are noted supraclavicular and axillary.

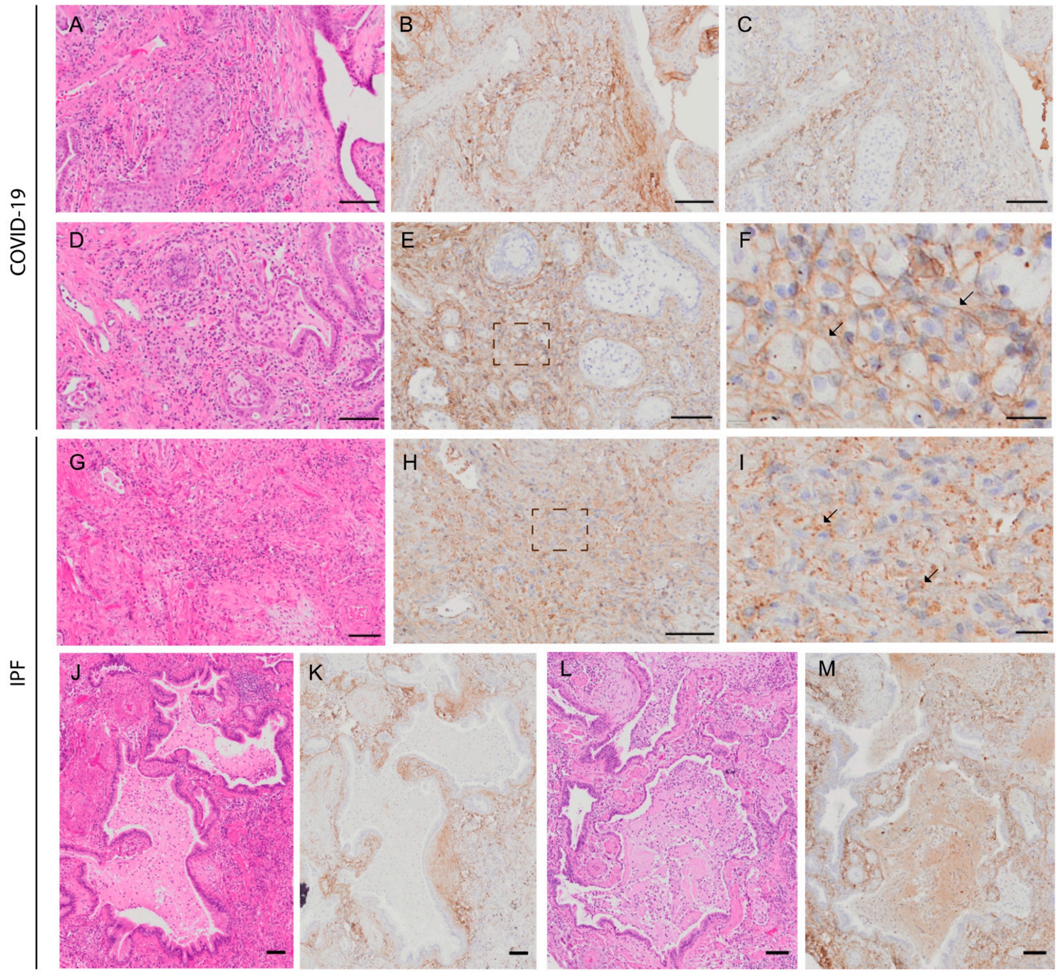

Figure S4. Pulmonary expression of DCN and POSTN in COVID-19 and IPF. A reversed pattern of periostin (POSTN) and decorin (DCN) could be seen in heavily remodeled areas (A, HE staining), where POSTN (B) was more intensely expressed in comparison to DCN (C). In regions with increased cell infiltration (D, G), DCN appeared incorporated in the ECM as elongated fibers (E, enlarged area F), while in IPF, DCN appeared fragmented (arrows) (H, enlarged area I). Honeycomb cysts in IPF (J, L) were enclosed by the expression of POSTN (K) and DCN (M).

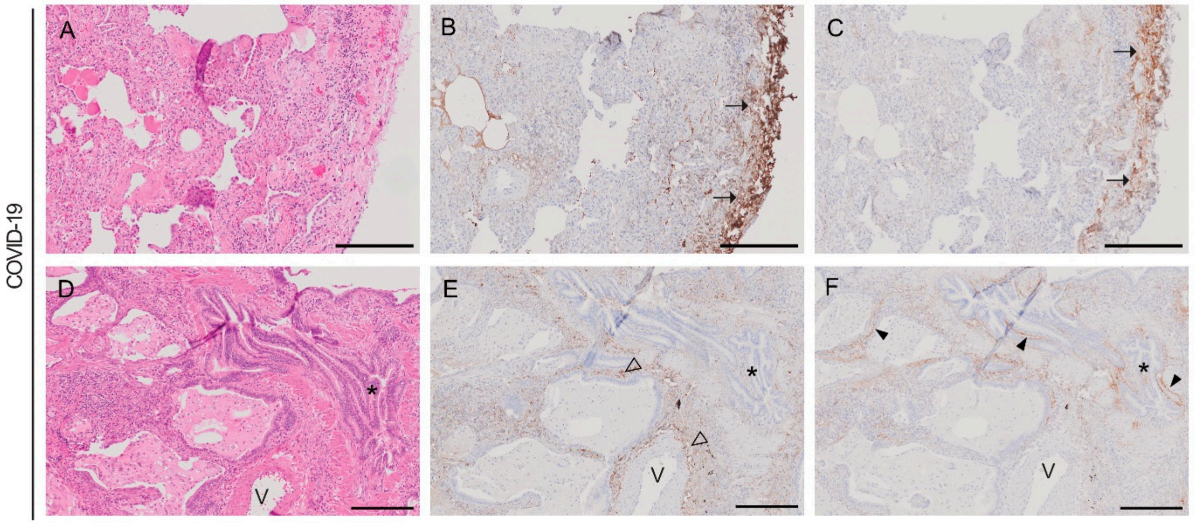

Figure S5. Pulmonary expression of DCN and POSTN in another COVID-19 patient. Both DCN (B, E) and POSTN (C, F) were observed in connective tissue of pleural and subpleural regions (B, C, arrows; HE in A, D). Furthermore, DCN was found in vascular adventitia (E, empty arrowheads), while POSTN was mainly localized to subepithelial regions of bronchioles and abnormal epithelial structures (F, full arrowheads). Scale bar = 250µm, * = bronchiole, v = vessel.
